# Supplementary figures and images for: Progranulin Protects against Hyperglycemia-Induced Neuronal Dysfunction through GSK3β Signaling
Source: Cells. 2023 Jul 7;12(13):1803. doi: 10.3390/cells12131803 (PMC10340575; doi:10.3390/cells12131803)

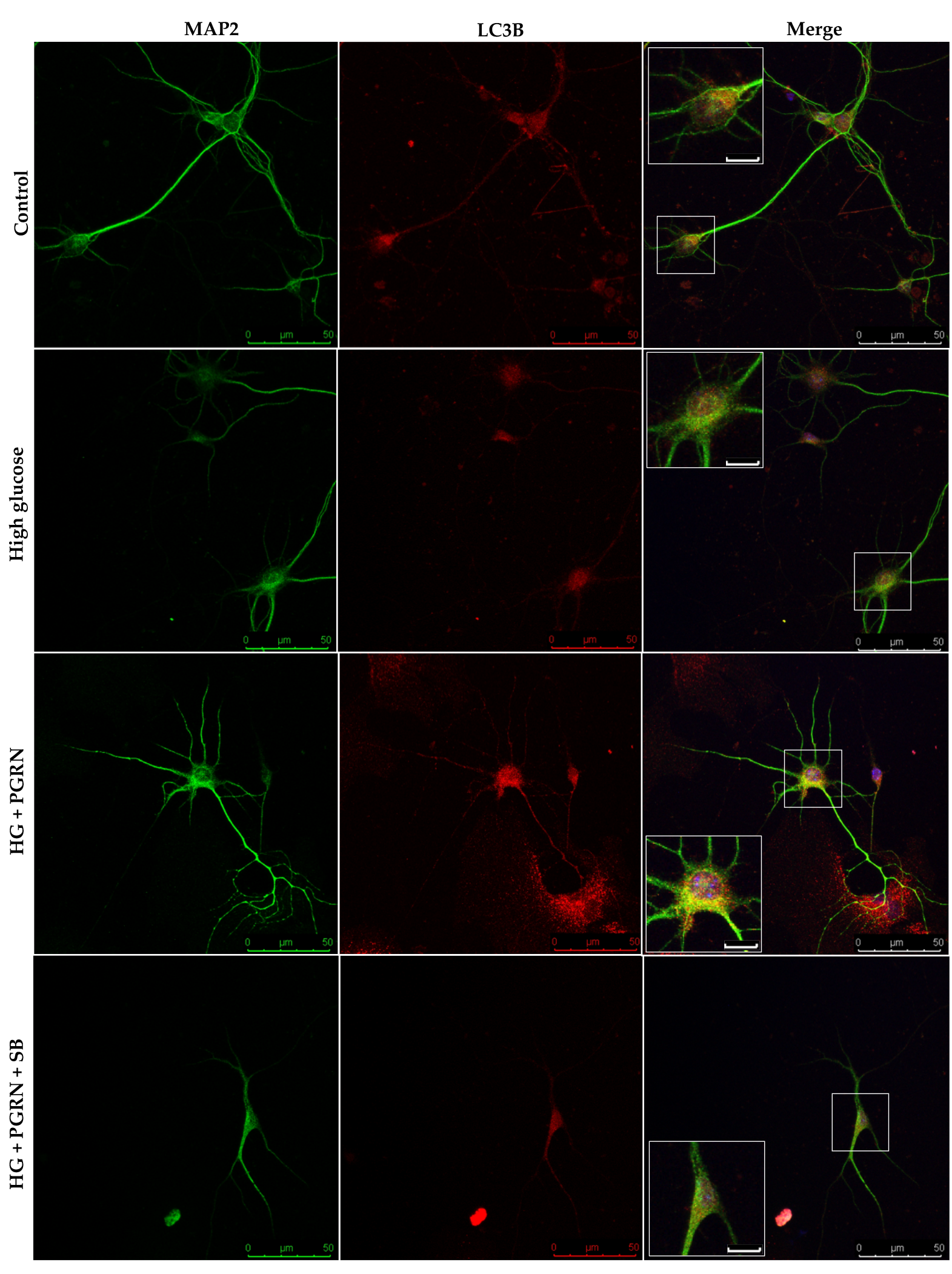

Supplement: Supplementary file 1 [file cells-12-01803-s001.zip › new supplemental figure 3.tif]

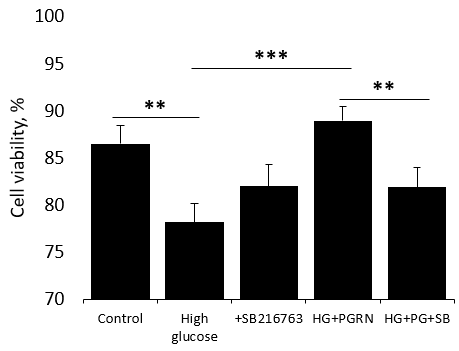

Supplement: Supplementary file 1 [file cells-12-01803-s001.zip › new supplementary figure 1.png]

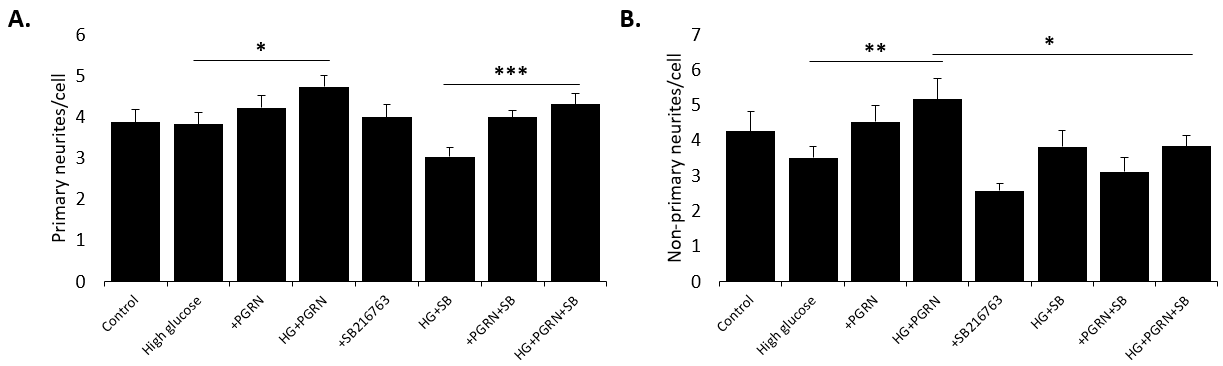

Supplement: Supplementary file 1 [file cells-12-01803-s001.zip › new supplementary figure 2.png]

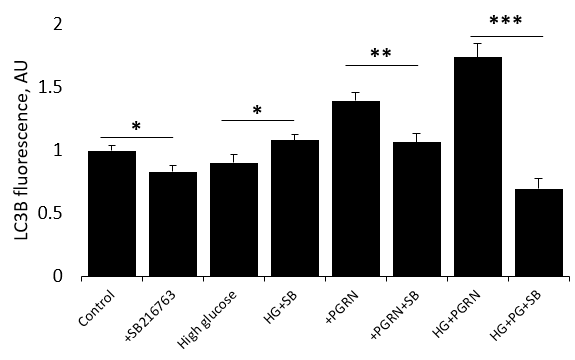

Supplement: Supplementary file 1 [file cells-12-01803-s001.zip › new supplementary figure 4.png]

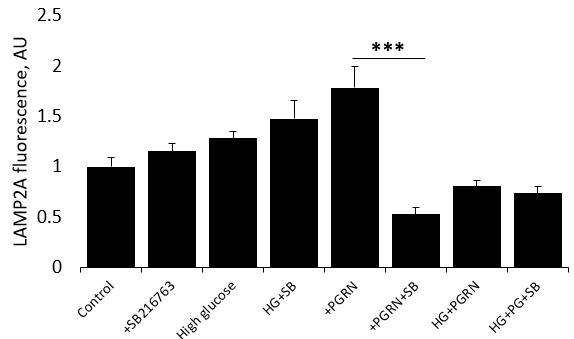

Supplement: Supplementary file 1 [file cells-12-01803-s001.zip › new supplementary figure 5.png]
